# Therapeutic potential of low-frequency transcranial magnetic stimulation in children with autism spectrum disorder: sensory and behavioral outcomes—a randomized trial

**DOI:** 10.3389/fpsyt.2026.1725598

**Published:** 2026-03-20

**Authors:** Hayfa Al-Ghabban, Fahad A. Bashiri, Shuliweeh Alenezi, Afaf El-Ansary, Asad Sabr, Laila Alayadhi

**Affiliations:** 1 Department of Anatomy and Physiology, Faculty of Medicine, College of Medicine, Imam Mohammed Ibn Saud Islamic University, Riyadh, Saudi Arabia; 2 Division of Pediatric Neurology, Department of Pediatrics, College of Medicine, King Saud University Medical City, King Saud University, Riyadh, Saudi Arabia; 3 Department of Psychiatry, College of Medicine, King Saud University, Riyadh, Saudi Arabia; 4 SABIC Psychological Health Research and Applications Chair (SPHRAC), Department of Psychiatry, College of Medicine, King Saud University, Riyadh, Saudi Arabia; 5 General Administration of Mental and Social Health, Assistant Agency for Hospital Services, Ministry of Health, Riyadh, Saudi Arabia; 6 Autism Center, Lotus Holistic Medical Center, Abu Dhabi, United Arab Emirates; 7 Autism Research and Treatment Center, Faculty of Medicine, King Saud University, Riyadh, Saudi Arabia; 8 Medicare Specialized Clinics, Riyadh, Saudi Arabia; 9 Department of Physiology, Faculty of Medicine, King Saud University, Riyadh, Saudi Arabia

**Keywords:** autism spectrum disorder, childhood autism rating scale, short sensory profile, social responsiveness scale, transcranial magnetic stimulation

## Abstract

**Background:**

Autism spectrum disorder (ASD) is a neurodevelopmental disorder that affects individuals across multiple life domains. Despite this, therapeutic options remain scarce, with none officially approved for its treatment. One recently explored treatment modality is repetitive transcranial magnetic stimulation (rTMS), a noninvasive neurostimulation intervention. This study examines the effectiveness of low-frequency rTMS in improving social, cognitive, and sensory function in individuals with ASD.

**Method:**

The study included 35 children aged 5 to 10 years who had been diagnosed with ASD. Participants were randomly assigned to two groups: 17 children received rTMS treatment twice weekly (active group), while the remaining 18 served as a waitlist (WTL) control group. Outcome measures were collected at baseline (preintervention) and 3 weeks after the completion of the 9-week rTMS intervention (postintervention).

**Results:**

Significant (*p* < 0.05) improvements in cognitive, social, and sensory functions were observed in the active group measures 3 weeks after the 9-week rTMS intervention (postintervention). This was evidenced by their Childhood Autism Rating Scale (CARS), Social Responsiveness Scale (SRS), and Short Sensory Profile (SSP) scores (27.47 ± 7.95, 71.59 ± 11.55, and 156.47 ± 20.17, respectively), with a highly significant reduction in sensory hypersensitivity (*p* = 0.001) compared to the WLT group, which presented more severe scores on CARS (35.89 ± 8.96), SRS (81.72 ± 7.68), and SSP (133.67 ± 11.65). Additionally, receiver operating characteristic (ROC) curves were analyzed. CARS, SRS, and SSP recorded poor areas under the curve (AUCs) in the active pre-rTMS group (0.557, 0.565, and 0.574, respectively), while all ROC curves showed significantly higher AUCs in post-rTMS (0.775, 0.753, and 0.825, respectively).

**Conclusion:**

The findings of this study suggest that low-frequency rTMS may have potential as an adjunctive intervention for specific ASD-related symptoms in social, cognitive, and sensory domains. However, further research is warranted to validate and expand these preliminary observations.

**Clinical Trial Registration:**

https://clinicaltrials.gov/study/NCT06524310, identifier NCT06524310.

## 1 Introduction

Autism spectrum disorder (ASD) is a neurodevelopmental condition characterized by chronic difficulties in social communication and interaction, as well as a refractory and repeated pattern of behavior, interests, or activities (1). Currently, therapeutic strategies for ASD target the core symptoms and related challenges. Pharmacological options, including atypical antipsychotics, are generally effective only in treating comorbidities rather than the primary symptoms of ASD (2, 3). Among nonpharmacological interventions, applied behavior analysis (ABA) is recognized for its broad effectiveness across multiple domains, while parent training has shown efficacy in managing irritability (3, 4). Moreover, cognitive behavioral therapy (CBT) has been found to improve ASD symptoms, and early interventions such as speech therapy demonstrate favorable outcomes for social and communication skills (5, 6). Although behavioral training is the most effective method, it is also the most costly and time-consuming.

Current research showed an emerging therapeutic approach using repetitive transcranial magnetic stimulation (rTMS) in the field of ASD. Multiple studies have explored its effects on various ASD symptoms and underlying neurophysiology. Both low- and high-frequency rTMS protocols have been investigated. Low-frequency rTMS has been shown to modulate autonomic measures and gamma-frequency oscillations in children with ASD (7, 8). Recent research has demonstrated that rTMS can lead to specific behavioral improvements, such as enhanced eye fixation.

Repetitive transcranial magnetic stimulation is a noninvasive treatment that involves placing a coil on the head and repeatedly applying an MRI-strength magnetic field. The magnetic field is generated by a rapidly pulsed current that penetrates the skull and stimulates the brain tissue, producing currents that may help normalize activity in the targeted location (9).

Given the prominent theory of abnormal synaptic plasticity and an imbalanced excitation/inhibition ratio in ASD, as well as the ability of rTMS to modify cortical excitability and plasticity, the potential of rTMS in ASD research is being investigated in laboratories worldwide (10). Repetitive transcranial magnetic stimulation is thought to induce long-term changes in the brain by modulating neural plasticity mechanisms and enhancing neuronal synaptogenesis (11), with several studies proposing that rTMS could normalize social and cognitive function in ASD by stabilizing aberrant neuroplasticity (12).

Glutamate transporters, particularly Excitatory Amino Acid Transporters (EAATs) on astrocytes and neurons, play a crucial role in regulating neuroplasticity by maintaining low extracellular glutamate levels, preventing overexcitation (excitotoxicity), and preserving synaptic specificity. They facilitate long-term potentiation (LTP) and depression (LTD) by limiting glutamate spillover and controlling extra synaptic receptor activity (13, 14).

rTMS has been shown to improve autistic-like behaviors in animal models by restoring excitatory/inhibitory synaptic balance and altering glutamate clearance, suggesting that it may help regulate synaptic plasticity. This study’s primary goal is to investigate and validate the efficacy of low-frequency rTMS in enhancing social, cognitive, and sensory abilities in children with ASD.

## 2 Materials and methods

### 2.1 Study design and setting

**Figure 1:**
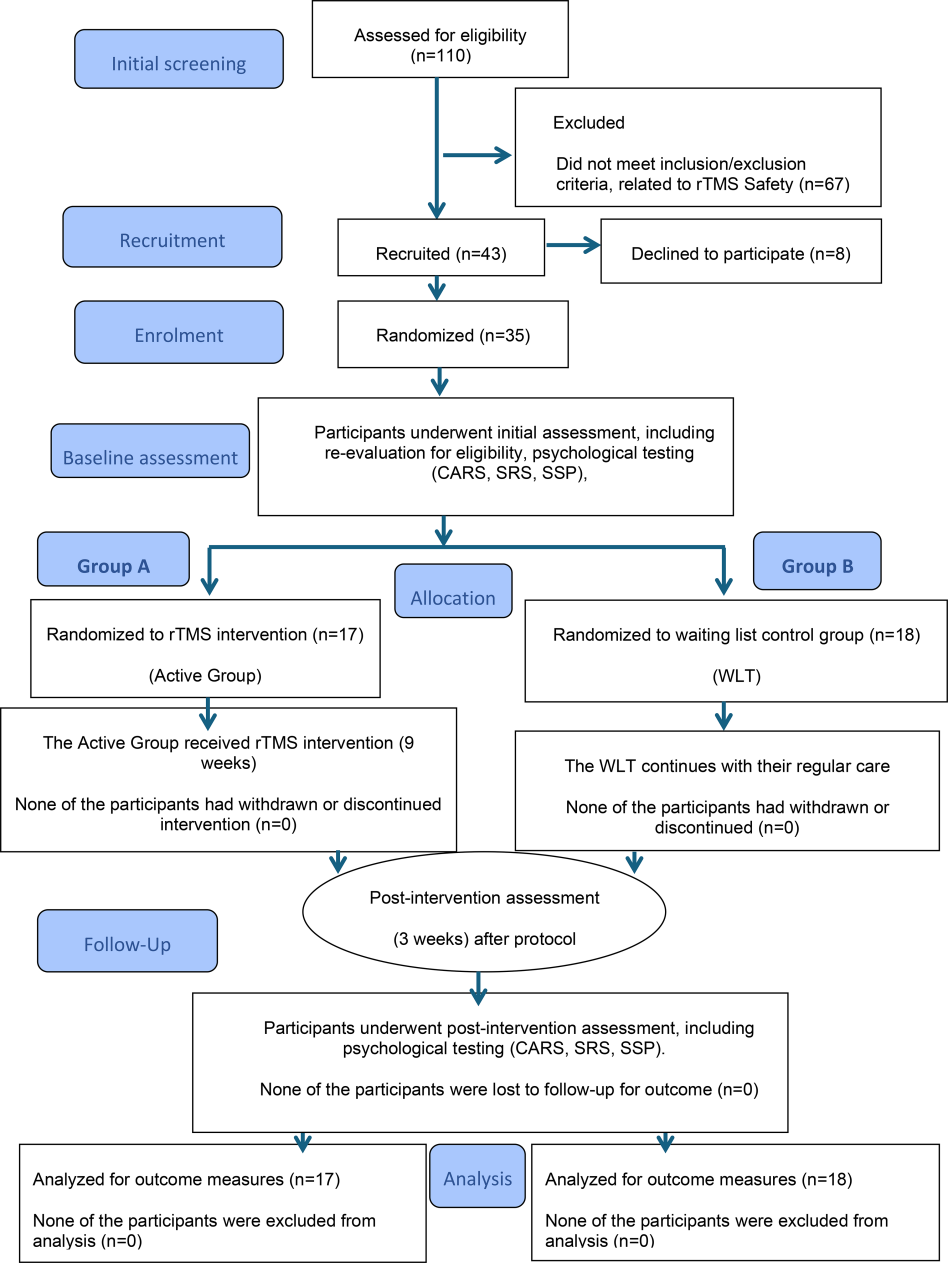
Trial FlowChart.

This prospective randomized controlled clinical trial was conducted and reported according to international standards (CONSORT) (**Figure 1**). The study took place from July 2024 to May 2025. The study protocol was reviewed and approved by the Institutional Review Board (IRB) of King Saud University, Riyadh, Saudi Arabia (IRB Approval No. 25/0047/IRB, project No. E-22-6654, on 06 March 2022, last renewed on 26 March 2025). The trial was registered with ClinicalTrials.gov PRS under the identifier (NCT06524310, on 30 June 2024; https://clinicaltrials.gov/study/NCT06524310). All procedures were performed in strict accordance with the ethical principles outlined in the Declaration of Helsinki (15). Written informed consent was obtained from the legal guardians of all participants before enrollment. The study was conducted at the Autism Research and Treatment Center (ARTC), Faculty of Medicine, King Saud University, the Medical City (KSUMC), and Medicare Specialized Clinics.

### 2.2 Participants and eligibility

A total of 35 children (25 boys, 10 girls, aged 5 to 10 years) with a confirmed diagnosis of ASD were recruited from the Childhood Autism Research and Treatment Center, King Saud University Faculty of Medicine. Inclusion criteria required a confirmed clinical diagnosis of ASD as defined by the Diagnostic and Statistical Manual of Mental Disorders, Fifth Edition (DSM-5). The diagnosis was validated either via a diagnostic report or through direct consultation with a diagnosing clinician, including a pediatric neurologist, child psychiatrist, or authorized psychologist.

Exclusion criteria in this study included the presence of comorbidities, such as neurological, psychiatric, or genetic conditions that might impact brain structure or function, including fragile X syndrome or tuberous sclerosis. Children with any known unstable medical condition or a diagnosed acute or chronic inflammatory condition were also excluded. Furthermore, individuals using high doses or combinations of psychotropic medications with significant seizure threshold-lowering potential were ineligible to participate.

Several exclusion criteria were established to comply with safety guidelines for rTMS (16), including a history of seizures, epileptiform activity, or repeated febrile seizures; the presence of any metallic implants within the cranium; a history of significant head trauma or loss of consciousness; and the presence of implanted electronic medical devices (e.g., pacemakers, cochlear implants).

Enrolled subjects had an IQ ≥ 70; this criterion was implemented to exclude cooccurring intellectual disability and to ensure that all participants possessed the cognitive capacity to comprehend study procedures and follow instructions during the intervention. Furthermore, as a critical safety measure, all participants were required to submit a recent electroencephalogram (EEG) report prior to enrollment for any underlying epileptiform activity and minimize the risk of seizure induction during rTMS sessions. It is important to clarify that both the IQ and EEG data were used exclusively for screening and eligibility purposes. Accordingly, these assessments were not conducted by the research team but were obtained from the participants’ existing medical records.

### 2.3 Randomization and group allocation

This investigation was conducted as a randomized controlled clinical trial with a waitlist control design. Sample size determination and study power were analyzed using the G*Power (version 3.1.9.7; Helsinki Heine University) software. Following the Cohen method, statistical analysis revealed that, at alpha (α) 0.05 with an effect size of 0.5 and a study power of 0.80 (80%), the required sample size was between 35 and 40 participants.

Group randomization was performed using a computer-generated randomization sequence with a block size of four (17). An independent statistician, uninvolved in participant recruitment or outcome assessment, developed the randomization sequence. The statistician placed the group assignments in sequentially numbered, opaque, sealed envelopes to guarantee allocation concealment. The envelopes were securely stored and opened in order by the principal investigator only after each participant completed baseline tests and provided informed consent. Participants who met the eligibility criteria were randomly assigned in a 1:1 ratio to the two groups. The intervention group (active group) (*n* = 17; median age: 8.0 years, interquartile range [IQR]: 3.50) received low-frequency rTMS immediately following baseline assessment, and the waitlist control (WLT) group (*n* = 18; median age: 6.5 years, IQR: 2.25) continued their usual treatment and was placed on a waiting list to receive the rTMS intervention after the study period. All participants in both study groups continued their medical and behavioral therapy for ASD. However, participants were instructed to maintain a consistent and unchanged medication regimen or behavioral therapy for at least 1 month prior to enrollment and throughout the trial.

Due to the nature of the WLT design and ethical considerations, blinding of participants and their guardians to group allocation was not possible. However, the clinicians performing the outcome assessments were blinded to the participants’ group assignments to minimize potential observer bias.

### 2.4 Intervention and adherence to protocol

Participants (active group) received rTMS using a figure-of-eight coil (Sentar^®^ Treatment Link, Malvern, PA, USA) connected to the NeuroStar TMS Therapy System Stimulator (Neuronetics Inc., Malvern, PA, USA). During the procedure, the electromagnetic pulses generated by rTMS targeted the dorsolateral prefrontal cortex (DLPFC), a site commonly used in most rTMS therapeutic protocols. The intervention comprised 18 sessions of active rTMS administered over 9 weeks. Treatments were administered twice per week; the first six sessions targeted the left DLPFC, the next six sessions targeted the right DLPFC, and the final six sessions were applied bilaterally, first over the left DLPFC and then the right DLPFC within the same session.

rTMS sessions were performed using the following protocol set: 1 Hz, 18 trains, 90% MT, 10-s train duration, 20-s intertrain interval, and a total of 180 pulses per session, lasting 6.18 min, using a figure-of-eight coil and a special sensory guard. This protocol was chosen to ensure both potential clinical benefit and enhanced safety. These parameters show a high degree of consistency with previous trials, which reported good tolerability and effective stimulation of cortical areas using low-frequency, short-duration protocols (8, 18, 19). Before each session, participants’ vital signs were monitored. Additionally, a checklist questionnaire was used to ensure that there were no major changes in the medications or behavioral treatment regimen the child was initially receiving prior to the intervention. During the intervention, participants were provided with well-fitted earplugs for auditory protection. Close monitoring was conducted to assess participant well-being and to report any side effects during the intervention. Furthermore, participants were observed immediately and 30 min after each session to identify any harms or side effects (e.g., headache or stimulation site discomfort). All sessions were performed under standardized conditions based on the guidelines provided by the rTMS therapy system manufacturer; participants were comfortably seated in a specialized armchair in a quiet environment. To ensure compliance during the intervention period, each participant was scheduled for rTMS sessions at a convenient time for their caregivers. Reminder text messages were sent to parents or caregivers before each session. An initial introductory interview was performed with each participant’s caregiver to emphasize the importance of session attendance and protocol compliance. However, any missed sessions were scheduled within the same week, and the reasons for missed sessions were documented. Documentation of each session was automatically generated by the rTMS-attached computer system and was regularly reviewed by the principal investigator.

### 2.5 Safety

Before enrollment, comprehensive screening was performed for all participants to confirm their eligibility for treatment, to ensure compliance with rTMS safety criteria, and to confirm the absence of any risk factors. Additionally, to enhance safety, rTMS was administered by a trained neuropsychiatric technician and an attending doctor capable of managing any adverse events. The stimulation intensity was adjusted to 90% of the motor threshold to minimize seizure risk. All adverse events were prepared to be thoroughly documented; however, no adverse events were recorded during the study. In the event of a serious occurrence, caregivers of all participants were instructed to seek immediate medical attention by presenting directly to the emergency department of King Saud University Medical City (KSUMC).

### 2.6 Assessments and outcome measures

Standardized behavioral assessments were administered to both groups at two distinct time points: at baseline before starting rTMS intervention (pre-rTMS) and 3 weeks after the completion of the 9-week rTMS intervention (post-rTMS). All assessments were conducted by board-certified clinicians with specific training and certification in their administration.

#### 2.6.1 Behavioral assessment

##### 2.6.1.1 Childhood autism rating scale, second edition

The Childhood Autism Rating Scale, Second Edition (CARS-2), was employed to quantify the severity of ASD symptoms (20). This 15-item observational scale, supplemented by caregiver report, evaluates core features of ASD (21, 22). Items are scored from 1 (within normal limits) to 4 (severely abnormal), with half-point increments allowed. Total scores are categorized as follows: 15–29.5 (minimal-to-no evidence of ASD), 30–36.5 (mild-to-moderate ASD), and ≥ 37 (severe ASD).

##### 2.6.1.2 Social responsiveness scale

The Social Responsiveness Scale (SRS) was used to provide a quantitative measure of reciprocal social behavior and autistic traits (23, 24). This 65-item rating scale assesses five domains: social awareness, social cognition, social communication, social motivation, and autistic mannerisms. Raw scores are converted to age- and gender-normed T-scores (mean = 50, SD = 10). T-scores ≥ 60 are considered clinically significant, with scores of 60–75 indicating mild-to-moderate impairment and scores > 75 indicating severe deficits in social responsiveness.

##### 2.6.1.3 Short sensory profile

The Short Sensory Profile (SSP), a 38-item caregiver questionnaire, was used to identify atypical sensory processing patterns. It assesses behavioral responses to sensory stimuli across seven domains (25). Item scores are summed and compared to age-based normative data to classify performance as “typical”, “probable differences”, or “definite differences”, indicating the severity of sensory modulation difficulties.

### 2.7 Statistical analysis

The results of the present study were analyzed using a commercially available software package, StatView (Abacus Concepts Inc., Berkley, CA, USA). The parametric data are presented as mean ± standard deviation (SD), while the nonparametric data are presented as median levels. A paired *t*-test was used to compare parametric data, and the Wilcoxon signed-rank test was used to compare nonparametric data (Kolmogorov–Smirnov test) pre- and post-rTMS. For all tests, a probability (*p*) of less than 0.05 was considered significant.

The area under the curve (AUC) is a widely used statistic for assessing the predictive capacity of biomarkers. Values near 1.00 indicate a highly predictive marker, whereas curves along the diagonal (AUC = 0.5) have no diagnostic relevance. When the AUC value is close to 1, the biomarker demonstrates the required specificity and sensitivity. To quantify the intervention’s effect, AUC was used to measure the magnitude of the treatment response by calculating the difference between pre- and postintervention AUC values.

The statistical analysis was conducted in two arms to comprehensively assess the intervention’s effects: first, a between-group comparison of outcome measures for the active and WLT groups at both baseline and postintervention; and second, a within-group longitudinal analysis comparing the active group’s baseline measures to their scores 3 weeks postintervention follow-up.

## 3 Results

**Table 1** shows the demographic characteristics of all participants in the study. **Table 2** demonstrates the differences between CARS, SRS, and SSP total scores in the active (pre-rTMS) and waitlist control (WTL) groups. It can be observed that the selection of both groups was appropriate, as neither group showed any significant differences in the three severity measures (CARS, SRS, and SSP).

**Table 1:** Participant demographics.

Demographic features	Active group	Waitlist control group (WTL)
Number	17	18
Gender (men: women)	11:6	14:4
Age (median, IQR, range)	8.00, 3.50 (5–10)	6.50, 2.25 (5–10)
Involvement in a rehabilitation or behavioral program	88.2%	88.5%
Pharmacological treatments (mean ± SD)		
Risperidone (0.5 mg/day)	0.22 ± 0.47	0.29 ± 0.43
Methylphenidate (0.3 mg/kg/day)	N/A	N/A
Atomoxetine (0.5 mg/kg/day)	N/A	N/A
History of medical or psychological comorbidities	N/A	N/A
Family history of mental or physical disability, including ASD	64.7%	77.8%
Siblings with ASD or other developmental delays	29.4%	29.4%

Data were calculated using the Chi-square test, Mann–Whitney *U* test (nonparametric data), and independent samples *t*-test (parametric data).

**Table 2:** Comparison between CARS, SRS, and SSP scores in the active (pre-rTMS) and WTL groups.

Parameter	Groups	*n*	Mean ± SD	*p*-value
CARS	Active group (pre-rTMS)	17	35.18 ± 10.35	0.610
	Waitlist control group (WTL)	18	37.06 ± 11.18	
SRS	Active group (pre-rTMS)	17	80.53 ± 12.39	0.423
	WTL	18	83.39 ± 8.16	
SSP	Active group (pre-rTMS)	17	126.94 ± 27.26	0.675
	WTL	18	123.39 ± 22.23	

Data were calculated using the independent samples *t*-test (parametric data).

CARS, Childhood Autism Rating Scale, Second Edition; SRS, Social Responsiveness Scale; SSP, Short Sensory Profile.

**Figure 2:**
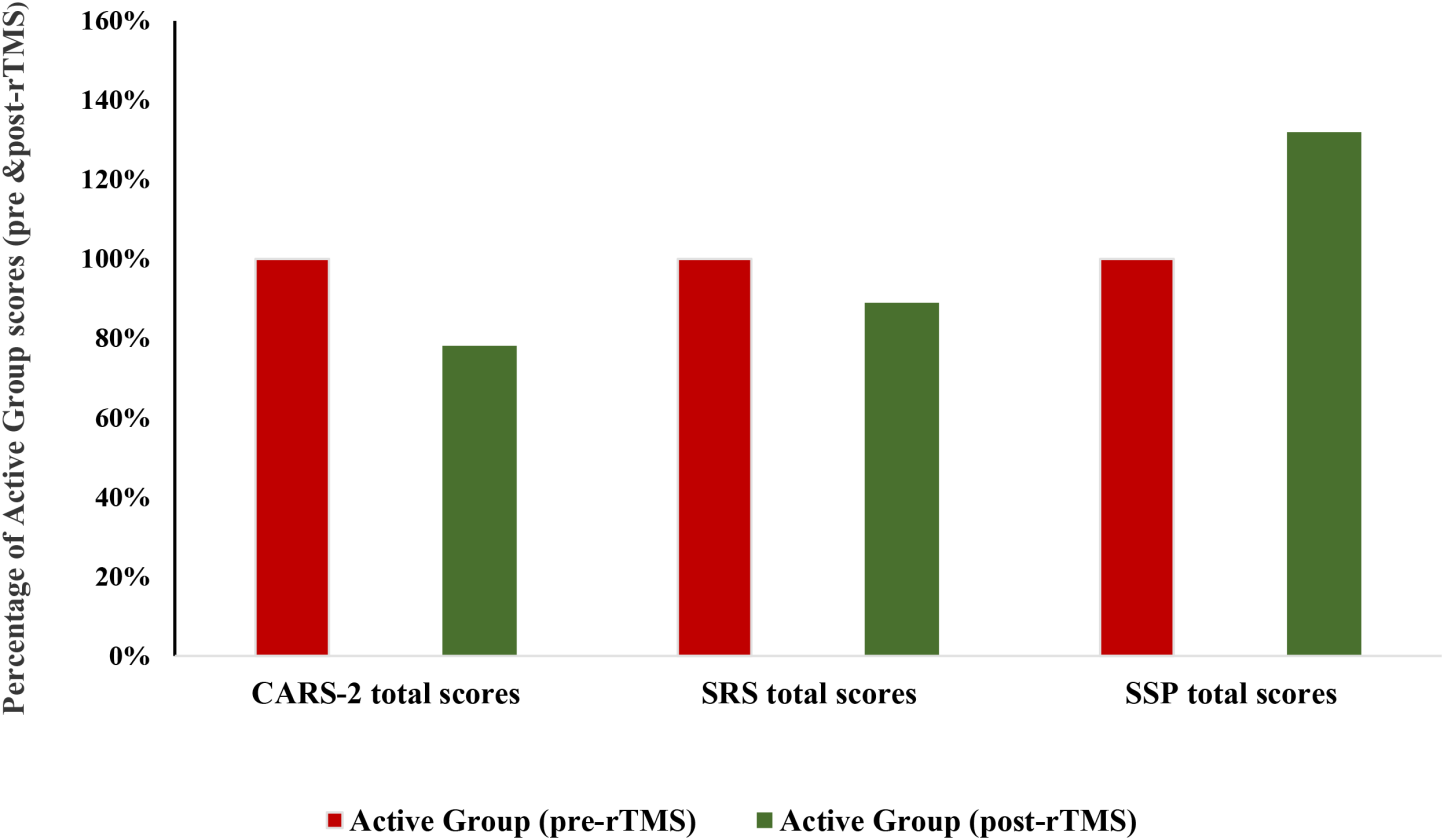
Percentage of mean for CARS, SRS, and SSP scores in the active group pre- and post-rTMS. CARS-2, Childhood Autism Rating Scale, Second Edition; SRS, Social Responsiveness Scale; SSP, Short Sensory Profile.

In contrast, **Table 3**, **4** show highly significant differences in the measured variables, with SSP demonstrating the most significant difference (*p* = 0.001) in the active group (post-rTMS) compared to WTL, as well as within the active group from pre- to post-rTMS. This pattern is clearly illustrated in **Figure 2**, which presents the percentage decrease or increase of CARS, SRS, and SSP in the active group (post-rTMS) relative to pre-rTMS assessments. While CARS and SRS, as measures of cognitive and social interaction impairment, decreased significantly by 21.92% and 11.1%, respectively, recording a CARS score of 27.47 ± 7.95 in the active group (post-rTMS), markedly lower than 35.18 ± 10.35 in the active group (pre-rTMS), SSP showed a much greater change, with a value of 23.26%.

**Table 3:** Comparison between CARS, SRS, and SSP scores in the active (post-rTMS) and WTL groups.

Parameter	Groups	*n*	Mean ± SD	*p*-value
CARS	Active group (post-rTMS)	17	27.47 ± 7.95	0.006^**^
	Waitlist control group (WTL)	18	35.89 ± 8.96	
SRS	Active group (post-rTMS)	17	71.59 ± 11.55	0.004^**^
	WTL	18	81.72 ± 7.68	
SSP	Active group (post-rTMS)	17	156.47 ± 20.17	0.001^***^
	WTL	18	133.67 ± 11.65	

Data were calculated using the independent samples *t*-test (parametric data).

CARS, Childhood Autism Rating Scale, Second Edition; SRS, Social Responsiveness Scale; SSP, Short Sensory Profile.

^*^p-value is less than 0.05, significant level (highly significant); ^**^p-value is less than 0.01, significant level (highly significant); ^***^p-value is less than 0.001, significant level (very highly significant).

**Table 4:** Comparison between CARS, SRS, and SSP scores in the active group pre- and post-rTMS.

Parameter	Groups	*n*	Mean ± SD	*p*-value
CARS total scores	Active group (pre-rTMS)	17	35.18 ± 10.35	0.004^**^
	Active group (post-rTMS)	17	27.47 ± 7.95	
SRS total scores	Active group (pre-rTMS)	17	80.53 ± 12.39	0.001^***^
	Active group (post-rTMS)	17	71.59 ± 11.55	
SSP total scores	Active group (pre-rTMS)	17	126.94 ± 27.26	0.001^***^
	Active group (post-rTMS)	17	156.47 ± 20.17	

Data were calculated using the paired samples *t*-test (parametric data).

CARS, Childhood Autism Rating Scale, Second Edition; SRS, Social Responsiveness Scale; SSP, Short Sensory Profile.

^**^p-value is less than 0.01, significant level (highly significant); ^***^p-value is less than 0.001, significant level (very highly significant).

**Figure 3:**
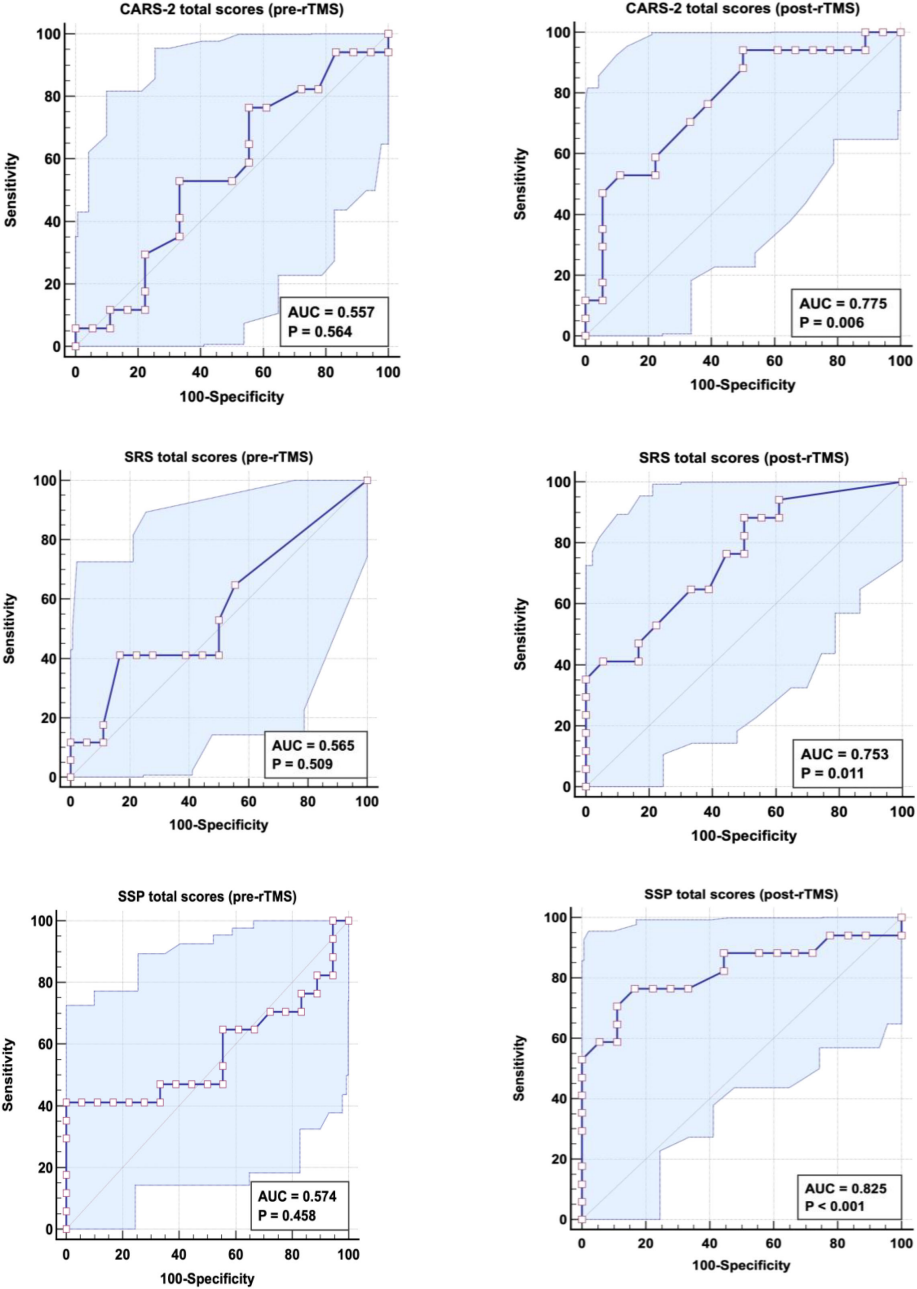
ROC curve for CARS, SRS, and SSP scores in the active group, both pre- and post-rTMS, using WTL as a reference group.

**Table 5** and **Figure 3** present the receiver operating characteristic (ROC) curve, AUC, specificity, and sensitivity of CARS, SRS, and SSP scores in the pre- and post-rTMS active groups, using WTL as the reference group. The three severity measures (CARS, SRS, and SSP) showed poor AUCs in the active group (pre-rTMS) (0.557, 0.565, and 0.574, respectively), whereas they exhibited substantially higher AUCs in the active group (post-rTMS) (0.775, 0.753, and 0.825, respectively).

**Table 5:** ROC results for CARS, SRS, and SSP scores in the active group pre- and post-rTMS according to WTL as a reference group.

Groups	AUC	Cutoff value	Sensitivity %	Specificity %	*p*-value	95% CI
CARS total score in the active group (pre-rTMS)	0.557	40.000	76.5%	44.4%	0.564	0.363–0.752
CARS total score in the active group (post-rTMS)	0.775	35.000	94.1%	50.0%	0.006	0.617–0.932
SRS total score in the active group (pre-rTMS)	0.565	76.500	41.2%	83.3%	0.509	0.371–0.760
SRS total score in the active group (post-rTMS)	0.753	83.500	88.2%	50.0%	0.011	0.594–0.913
SSP total score in the active group (pre-rTMS)	0.574	148.500	41.2%	100.0%	0.458	0.370–0.777
SSP total score in the active group (post-rTMS)	0.825	142.000	76.5%	83.3%	0.001	0.675–0.975

CARS, Childhood Autism Rating Scale, Second Edition; SRS, Social Responsiveness Scale; SSP, Short Sensory Profile.

## 4 Discussion

ASD is a complex and heterogeneous neurodevelopmental condition characterized by persistent difficulties in social communication and interaction, alongside restricted, repetitive patterns of behavior, interests, or activities (26, 27). Behavioral and pharmacological approaches are commonly employed to treat individuals with ASD. Although behavioral training is the most efficacious intervention, it is also the most financially burdensome and time-consuming. Furthermore, drug therapies are typically more effective in treating comorbidities rather than the primary symptoms of ASD (2).

The current study investigated whether rTMS has a potential therapeutic benefit or is ineffective in treating core symptoms of ASD. Comparison of symptom intensity before and after 18 rTMS sessions revealed a substantial difference and improvement in clinical symptoms, as measured by CARS, SRS, and SSP, which assess different severity levels in children with ASD (32).

Research has indicated that low-frequency rTMS (≤ 1 Hz) promotes inhibition of activated cortex; 1 Hz was used as the stimulation frequency (28). The lower the frequency of rTMS, the lower the risk of seizures (8). The current investigation used both stimulatory and inhibitory < 1 Hz frequencies, as in previous studies (8, 29, 30). Excitation/inhibition imbalance has been proposed as the primary factor influencing neuroplasticity anomalies in neurodevelopmental diseases, including ASD (31), and the pathophysiology of ASD has been linked to a deficiency in inhibitory neurotransmission.

The current study focuses on a younger cohort based on the principle of developmental neuroplasticity, which theorizes that the brain’s capacity for change and adaptation is higher during earlier life stages, suggesting that interventions may be more effective when administered during these sensitive periods (32, 33). Additionally, by using a narrow age range (5–10 years), this study aimed to create a more homogeneous sample, helping to reduce the impact of age-related differences on rTMS outcomes. Our results indicate that there were no significant differences in the baseline assessment of CARS, SRS, and SSP between the active and WLT groups (pre-rTMS).

In contrast, significant differences were found in CARS, SRS, and SSP between the active and WTL groups (post-rTMS), as well as within the active group from pre- to post-rTMS. Our study shows that the significant decrease in CARS score, as a measure of cognitive ability in ASD, is supported by a meta-analysis, which found that rTMS therapy in patients with mild cognitive impairment (MCI) and early Alzheimer’s disease (AD) can significantly improve not only global cognitive ability but also memory, executive function, and language when compared with the control group (34). Most rTMS parameter settings can significantly enhance global cognitive performance (31). Additionally, our data are in good agreement with the study by Abd et al. (35), which reports promising results following rTMS in the neuropsychological development of pediatric subjects with ASD.

In the current study, without physiological measurements, these clinical findings cannot be clearly attributed to particular brain pathways. However, the significant improvement in measures targeting different severity levels in children with ASD aligns with literature-based models of glutamate regulation in ASD. Although clinical evidence remains limited and further studies are required, the management of glutamate homeostasis is gaining interest as a potential critical target for controlling brain illnesses, including ASD (35). The brain’s ability to resist excitotoxicity depends on glutamate clearance, which is primarily regulated by astrocytic transporters (GLT-1/EAAT2). Impaired clearance is sometimes associated with neurodevelopmental disorders. Better clinical outcomes, such as lower CARS scores, may be linked to higher glutamate clearance in the context of ASD. This theoretical model could help to explain the significant decrease in CARS observed in the active group (post-rTMS) compared to the measures obtained for the active group (pre-rTMS), suggesting that rTMS may counteract the reduction of GLT-1 on astrocytes and potentially reduce glutamate accumulation in the synaptic cleft. This reduction might, in turn, improve synaptic plasticity and mitigate neuronal apoptosis (14). This could find support in the study of Li et al. (36), which shows the correlations between CARS scores and glutamate concentrations in individuals with ASD. High glutamate levels lead to neuronal dysfunction through oxidative stress, mitochondrial damage, and uncontrolled calcium influx (37). This disturbance of synaptic pruning and neural network construction may hinder neural connections and brain circuit growth. Long-term excitotoxicity can endanger neuronal survival and promote neurodevelopmental abnormalities, suggesting that these alterations may underlie the distinctive behavioral and cognitive challenges associated with ASD (38, 39). Therefore, the glutamatergic and plasticity-related mechanisms discussed here should be considered theoretical models for future investigation in multimodal research.

The remarkable improvement of social interaction in the active (post-rTMS) compared to the control or active group (pre-rTMS) is reflected in the lower recorded SRS score. This finding aligns with Yuan et al. (10), who reported noticeable enhancements across a spectrum of scales in stereotyped behavior, repetitive behavior, and verbal social domains following rTMS intervention. The results are further supported by the studies of Abd et al. (35) and Darwish et al. (40), which found that, when comparing pre- and post-rTMS outcomes based on clinical severity of ASD according to DSM-5, the improvement in social communication was statistically significant (*p*-value = 0.001).

The recorded improvement of SSP scores in the active group (post-rTMS) compared to the control or active group (pre-rTMS) is consistent with previous work, which demonstrates that the effectiveness of rTMS stimulation in treating comorbid sleep disorders in children with ASD is partially mediated by the treatment of sensory abnormalities (41, 42).

Hazen et al. (43) suggest that combining conventional therapies and 16 sessions of low-intensity TMS (Li-TMS) for individuals with ASD resulted in significant clinical progress, specifically in maturation development as measured by BDI. In addition, the use of low-intensity magnetic fields may allow for safer pulse delivery in pediatric subjects, as no side effects were reported in this study. These findings support the use of low-intensity TMS in the current study (1 Hz, 18 trains, 90% MT, 10-s train duration, 20-s intertrain interval). Sensory processing problems, including hyper-/hyposensitivity to auditory, visual, and tactile inputs, have been identified as a feature of restricted, repetitive patterns of behavior, interests, or activities (41).

Autism spectrum disorder patients frequently exhibit hypersensitivity to auditory stimuli, which may impede behavioral adaptation (41, 42). The current study’s considerable improvements in CARS, SRS, and SSP in the active group (post-rTMS) compared to the WLT or active group (pre-rTMS) may indicate a critical role of impaired glutamate clearance in the etiology of ASD. This is supported by the work of Ikeda et al. (44), who reported that rTMS has been used to treat dementia, obsessive–compulsive disorder (OCD), and certain schizophrenia symptoms, and it appears to ameliorate symptoms by enhancing transporter-mediated glutamate clearance.

The CARS, SRS, and SSP are reliable screening and diagnostic instruments for ASD, with ROC studies routinely yielding high AUC values and sensitivity/specificity, particularly when distinguishing autism from other developmental impairments. They also demonstrate strong predictive power in monitoring improvements in ASD, especially when assessing treatment effectiveness over time. Our results present the ROC curves, AUCs, specificity, and sensitivity. Although the three scores (CARS, SRS, and SSP) reported low AUCs in the active group (pre-rTMS), showing no discrimination between the WLT and active group before the rTMS intervention—a point that could be considered a strength of the study—the effectiveness of rTMS is evident from the remarkable increase in the AUCs of the three scores. The three severity measures (CARS, SRS, and SSP) had low AUCs in the active group (pre-rTMS) (0.557, 0.565, and 0.574, respectively) but achieved substantially higher AUCs after rTMS (0.775, 0.753, and 0.825, respectively).

## 5 Conclusion

This study suggests that repeated bilateral low-frequency TMS sessions over the left and right DLPFC may be a useful supplementary intervention, as they have shown significant improvement in certain ASD-related symptoms in the social, cognitive, and sensory domains. The current findings should be viewed as hypothesis-generating observations intended to guide future research rather than as proof of neurobiological repair or treatment efficacy. These results should be interpreted cautiously in light of the study’s limitations. To validate and build upon these initial findings, especially in the Saudi Arabian context, more research is required, including bigger and more diverse populations as well as prolonged follow-up.

### 5.1 Limitations and future research

This study has several limitations that should be acknowledged. The sample size was relatively small and drawn from a single center, which may limit generalizability. Limitations related to participant characterization should also be considered when interpreting the findings. There was an overrepresentation of male participants compared to female participants, which may reflect the nature of the disorder. Although ASD represents a single diagnostic category, considerable individual variability exists in clinical presentation, cognitive profile, and adaptive functioning, potentially influencing responsiveness to intervention. While an IQ ≥ 70 was an inclusion criterion based on previous clinical records, standardized IQ assessments were not administered as part of the study protocol. The absence of these data limits the participant’s cognitive profile. In addition, the potential effects of concomitant medication use were not systematically accounted for. Although an EEG was required for safety screening, it was not collected for subsequent analysis. Furthermore, the use of a waiting list rather than a sham-control group, together with the inability to blind participants and caregivers, increases the risk of expectancy and placebo effects. The limited diversity of participants, driven partly by rTMS safety criteria, constrains generalizability. Finally, the cultural context of a Saudi Arabia-only cohort may limit applicability to other populations. Further investigation, including multicenter clinical trials with larger and more diverse cohorts, multiple follow-ups, and the inclusion of adolescents, young adults, and individuals with ASD and intellectual disabilities, is strongly recommended. Future studies should also examine expanded protocols (e.g., higher stimulation intensity or longer treatment duration) and incorporate multimodal outcome measures (e.g., neuroimaging or neurophysiological testing) to better understand efficacy and the mechanisms of change.

## Data Availability

The original contributions presented in the study are included in the article/supplementary material. Further inquiries can be directed to the corresponding author.

## Glossary

ABA: applied behavior analysis
AD: Alzheimer’s disease
ASD: autism spectrum disorder
AUC: area under the curve
CARS-2: Childhood Autism Rating Scale, Second Edition
CBT: cognitive behavioral therapy
DG: dentate gyrus
DLPFC: dorsolateral prefrontal cortex
DSM-5: Diagnostic and Statistical Manual of Mental Disorders, Fifth Edition
E/I: excitation to inhibition
EAATs: excitatory amino acid transporters
EEG: electroencephalogram
ELISA: enzyme-linked immunosorbent assay
GABA: gamma-aminobutyric acid
GLT-1: glutamate transporter-1
IQ: intelligence quotient
Li-TMS: low-intensity transcranial magnetic stimulation
LTD: long-term depression
LTP: long-term potentiation
MCI: mild cognitive impairment
NKCC1: Na–K–Cl cotransporter
OCD: obsessive–compulsive disorder
ROC: receiver operating characteristic
rTMS: repetitive transcranial magnetic stimulation
SRS: Social Responsiveness Scale
SSP: Short Sensory Profile
TMS: transcranial magnetic stimulation
WLT: waitlist control
